# Bedside teaching in medical education: a literature review

**DOI:** 10.1007/s40037-013-0083-y

**Published:** 2013-09-19

**Authors:** Max Peters, Olle ten Cate

**Affiliations:** 1 Utrecht University, University Medical Center Utrecht, Heidelberglaan 100, CX 3584 Utrecht, the Netherlands; 2 Center for Research and Development of Education, University Medical Center Utrecht, Utrecht, the Netherlands

**Keywords:** Bedside teaching, Decline, Clinical skills, Satisfaction, Future directions/solutions

## Abstract

Bedside teaching is seen as one of the most important modalities in teaching a variety of skills important for the medical profession, but its use is declining. A literature review was conducted to reveal its strengths, the causes of its decline and future perspectives, the evidence with regard to learning clinical skills and patient/student/teacher satisfaction. PubMed, Embase and the Cochrane library were systematically searched with regard to terms related to bedside teaching. Articles regarding the above-mentioned subjects were included. Bedside teaching has shown to improve certain clinical diagnostic skills in medical students and residents. Patients, students/residents and teachers all seem to favour bedside teaching, for varying reasons. Despite this, the practice of bedside teaching is declining. Reasons to explain this decline include the increased patient turnover in hospitals, the assumed violation of patients’ privacy and an increased reliance on technology in the diagnostic process. Solutions vary from increasingly using residents and interns as bedside teachers to actively educating staff members regarding the importance of bedside teaching and providing them with practical essentials. Impediments to bedside teaching need to be overcome if this teaching modality is to remain a valuable educational method for durable clinical skills.

## Introduction

‘To study the phenomena of disease without books is to sail an uncharted sea whilst to study books without patients is not to go to sea at all’, and: ‘Medicine is learned by the bedside and not in the classroom’ are quotes of the famous Sir William Osler [1]. William Osler (1849–1919) was, in the century after Herman Boerhaave, among the greatest promoters of bedside teaching as a mode of medical education and his words are still valid more than a century later [1, 2].

Traditionally, bedside teaching has always been seen as a primary teaching modality in which most aspects of clinical practice can be demonstrated and trained. It was widely used across medical schools in the first half of the previous century, and was estimated to represent as much as 75 % of all clinical training in the 1960s [3]. The recent explosion of imaging and laboratory testing has decreased its use [4]. Today’s estimates range from 8–19 %, if at all present in medical training [3, 5]. Bedside teaching has been described as one of the ideal clinical teaching modalities, in which history taking and physical examination skills, together with professional attitude, can be combined to provide a holistic approach in the diagnostic process and in patient care. Students and residents are found to be motivated to engage in clinical reasoning and problem-solving if their preceptor, acting as a role model, provides adequate demonstration and guidance [3, 6–8]. Furthermore, a thorough and correct history and physical examination have been shown to provide the correct diagnosis in 73 % of cases, on average. For certain problems, this percentage can be over 90 % [9].

Several other skills essential in patient contact can, for a great part, best be learned at the bedside. Most importantly communicating effectively with real patients, but also medical ethics (for example discretion regarding sensitive subjects) and adequately obtaining a structured history without the use of extensive medical terminology [5].

However, when looking for instructions how to conduct bedside teaching, disappointingly little is found in textbooks. We scrutinized 17 recent books on medical education to look for practical advice on how to carry out bedside teaching [10–26] (Table 1). Only two listed ‘bedside teaching’ in their index, 7 had a chapter on patient-based clinical teaching in the ward or in the outpatient clinic; 10 had no such chapter. Nine books did not give any information on patient-based clinical teaching. Those that did spent 3–12 % of their pages on it; part of this was on bedside teaching. Books that stand out with clear educational instruction are Peyton (1998) [10], Alguire et al. (2008) [16], Dent and Harden (2009) [19] and Rubenstein and Talbot (2013) [26]. Clearly, bedside teaching is not treated as being very central to medical education by many authors in the field. We wondered what the reasons are for the decline in the focus on this approach to teaching in medicine and if evidence is available regarding the educational value of bedside teaching.

**Table 1:** Information on bedside teaching found in textbooks

Book	‘Bedside’ or BST mentioned in index	Chapter devoted to BST	Chapter on patient-based teaching in ambulatory care	Pages and percentage of total spent on BST*	Pages and % of total spent on patient-based in ambulatory clinic
(Jeffries and Huggett, 2010)	No	Yes	No	11 (5 %)	O
(Swanwick, 2010)		Yes		10 (2.5 %)	0
(Dornan et al. 2011)	No	No	No	0	0
(Salerno-Kennedy and O’Flynn, 2010)	No	No	No	2 (1.5 %)	2 (1.5 %)
(Delany and Molloy, 2009)	No	No	No	0	0
(Bland et al. 2007)	No	No	No	0	0
(Skeff and Stratos, 2010)	Yes	No	No	0	O
(Alguire et al. 2008)	No	No	Yes	0	15 (8 %)
(Carter and Jackson, n.d.)	No	No	No	0	0
(Harden and Laidlaw, 2012)	No	Yes		5 (2 %)	
(Peyton, 1998)	No	Yes	Yes	15 (8 %)	8 (4 %)
(Fish and Coles, 2005)	No	No	No	0	0
(Rubenstein and Talbot, 2013)	No	No	Yes	0 (0 %)	+/−20 (12 %)
(Dent and Harden, 2009)	Yes	Yes 6 pp	Yes 9 pp	6 (1.5)	9 (2)
(Amin and Khoo, 2003)	No	No	No	0	0
(Newble and Cannon, 2001)	No	No	No	0	0
(Gunderman, 2006)	No	No	No	0	0

*BST* Bedside teaching

Explanatory information: information on how to do BST. Less than half a page is counted 0

Case-based teaching (teaching after the trainees had seen a patient) is not considered BST

In this literature overview we have attempted to evaluate the reasons for the decline in bedside teaching and to depict current evidence in the discussion on the effects of bedside teaching on learning, including the views of the parties involved and possible directions for the future.

## Methods

Our initial question focused on what bedside teaching can offer to the acquisition of durable clinical skills in addition to other teaching modalities. PubMed, Embase and the Cochrane library were searched by one of the authors for articles concerning bedside teaching and the learning of clinical skills (Table 1). Synonyms were used for the domain “medical students/residents/teachers” and the determinant “bedside teaching” Table 2.

**Table 2:** Search syntax for PubMed, Embase and Cochrane

Database	Syntax (08-01-2013)	Hits
PubMed (TIAB)	(Medical students OR Students OR Residents OR Interns OR Medical residents OR Medical interns OR Medical teachers OR Medical tutors) AND (Bedside teaching OR Bed-side teaching OR teaching at the bedside OR bedside education OR bedside-education OR bedside demonstration OR bedside training)	584
Embase TI, AB	The above-mentioned search: all search terms in title and abstract	7
Cochrane AB, TI, KW	The above-mentioned search: all search terms in title and abstract	34
Total		625

*AB* abstract, *KW* keywords, *TI* title

**Fig. 1:**
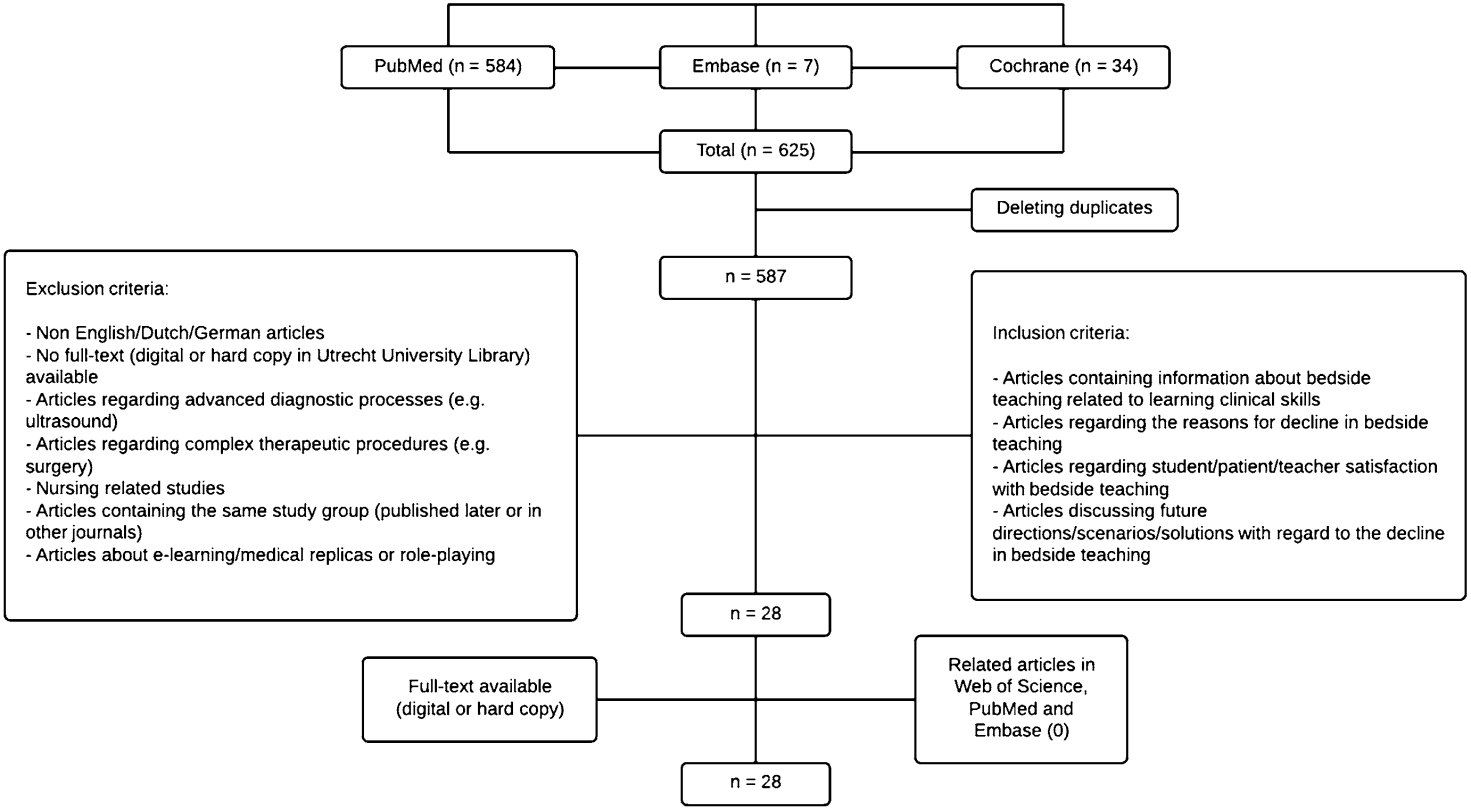
Flow-chart depicting the search strategy

Relevant articles were screened with regard to title and abstract and if necessary in full text. Inclusion and exclusion criteria were applied to obtain articles regarding the evidence of learning clinical skills, reasons for the decline in bedside teaching, future directions and attitudes/opinions of persons involved (Fig. 1). Both authors reviewed the resulting abstracts and concluded that a total of 29 articles contained useful information related to the above-mentioned subjects regarding bedside teaching [3, 5–8, 27–50]. All articles were available in full text (either digitally or in hard copy in the University Utrecht library). No relevant related articles were found in the Web of Science or in the related articles in PubMed or Embase.

Because contemporary evidence regarding the added value of bedside teaching is relatively scarce and most articles were of a descriptive nature, methodological inclusion and exclusion criteria were not broadly applied (Fig. 1).

## Results

### Reasons for the decline in bedside teaching and consequences

A frequently encountered reason for the decline in bedside teaching is the changing nature of teaching hospitals, especially the shortened admittance of patients, which increases the workload of physicians while decreasing the potential suitability of patients for bedside rounds [8, 46]. The practical hindrances for teaching mentioned vary from time constraints, patients not being in bed and noise on the ward, but also poor clinical skills and knowledge of students [8].

Discrepancies are observed in how bedside teaching is viewed by medical teachers and patients. The majority of patients appear to appreciate bedside teaching because of the extra time and insight given to their medical situation. However, physicians seem to favour this teaching method far less, especially younger physicians, afraid of it being demeaning and burdening to patients [5, 8, 31]. Bedside rounds are frequently substituted by conference room presentations. One of the most frequently proposed reasons for this shift is the transition from patient-oriented medicine to an increasing reliance on technology and laboratory testing in the diagnostic process [5, 31, 39, 46]. The conference room provides a comfortable alternative to the bedside. Here, the teacher is fully in control, able to guide the direction of the discussion within his own area of expertise, without (unexpected) interference from the patient. Furthermore, results from advanced diagnostic procedures can be more readily viewed and larger numbers of students can be taught at the same time in this manner [5].

A vicious circle seems to result: the less one goes to the bedside and the more one depends on the technical aspects of the diagnostic process, the more uncomfortable one eventually becomes at the bedside, leading to a further decrease in time spent at the bedside [46]. There seems to be a decline in physical diagnostic skills in junior doctors alongside the decline in the time spent on bedside teaching in the medical curricula [31]. Although a definitive causal connection cannot be made solely on the basis of this observation, it stands to reason that a decline in examination and history taking skills is at least partly related to a decline in the direct teaching of these essential medical competencies. Is there evidence for this statement?

### Bedside teaching and clinical skills

The initial search focused on the available evidence related to the learning of clinical skills with regard to bedside teaching. Many of the arguments encountered seem to be based on rational assumptions. Certain aspects of the physical examination can hardly be learned any other way than with real-life practice. The patient encounter is still viewed by some as the ideal place in which personal and disease-specific aspects can be practised (such as learning the sensation of hepatosplenomegaly) [7, 38]. This argument can be expanded to other parts of the physical examination and to history taking as well [4]. With these modalities it could be theorized that they can be simulated with actors or medical replicas. Even though simulations provide a reasonable approximation of real pathology, the actual clinical encounter itself might not be fully mimicable [7, 51]. Elaborating illness scripts, the structured mental models of diseases in which the presentation of patients together with relevant knowledge about clinical conditions is connected with clinical reasoning, requires experiences with real patients [51, 52].

However, simulation provides a variety of advantages over actual patients being studied. For particular parts of history taking and physical examination, simulation proves to be just as effective as real patient contact [51]. Furthermore, simulation by actors provides the possibility of direct feedback, students feel more comfortable with their mistakes and simulations can be repeated and planned in a more structured and flexible way compared with practice with patients [51]. Notwithstanding these advantages, certain pathological conditions are impossible to simulate genuinely, which leaves only the actual patient encounter as the primary learning modality.

In conclusion, rational arguments in favour of bedside teaching are justified to a certain extent on theoretical grounds. But what is the evidence in the literature regarding this potential added value of bedside teaching?

### Evidence

Prospective randomized studies regarding bedside teaching are not abundant in the literature. Furthermore, not much seems to be written about bedside teaching after 2008. In 1983, Cooper showed that physical examination skills and history taking (with regard to gastrointestinal pathology) practised with real patients in addition to healthy subjects in teaching sessions, resulted in significantly better scores on an OSCE (objective structured clinical examination) for fourth-year medical students [28]. There was a trend for better scores related to group size (smaller groups received a better OSCE score). Other evidence comes from the training of residents in cardiology [30, 32, 45]. When comparing residents from the United Kingdom, Canada and the United States with regard to the correct identification of 12 types of heart murmurs, the overall scores in all three countries were poor (mean 20–25 % for all three countries). It appeared that Canadian residents performed slightly (but significantly) better and that British residents improved the most during their training. The scores correlated with the amount of practical teaching of heart murmurs provided during medical school and residency [53].

Favrat et al. [30] also evaluated cardiac auscultation, but this time solely at the bedside, stating that this would better reflect the added value of actual bedside teaching. Two experienced cardiologists and 20 residents in internal medicine or family practice listened to 33 cardiac events at the bedside across 13 different patients. The 13 possible diagnoses were predefined by a cardiology expert and confirmed by echocardiographic evaluation. Ten residents subsequently enrolled in a five-month training programme with weekly 45-minute cardiac auscultation training sessions. The cardiologists correctly identified 69 % of cardiac murmurs and made a correct diagnosis in 62 % of cases. In comparison, without training, 40 % of murmurs were correctly identified by the residents, and they subsequently made a correct diagnosis in only 21 % of the cases. After the training programme, the identification of murmurs did not increase, but a correct diagnosis was made in 35 % of cases, reflecting an increase of 66 % compared with baseline. Aortic insufficiency and aortic stenosis were the modalities with the greatest increase in correct diagnosis. No relationship was seen between self-assessment of the residents and the amount of correct diagnoses they made.

Another, more recent, study in the cardiology field was done by Sverdrup et al., in which third-year medical students randomly received a (short, four-hour) course of computer-simulated heart sounds or additional bedside training [45]. This study found no difference between the two groups in recognizing pathological heart sounds.

Other evidence of the added value of bedside teaching comes from a study done with sixth-year medical students from Germany doing a four-month elective in neurology [35]. In this study, two groups were analyzed in which the control group received the normal theoretical curriculum during their elective. In the intervention group the focus was shifted from didactic teaching of neurological subjects to emphasis on history taking, physical examination and other methods to diagnose neurological disease. This approach was supplemented with daily bedside teaching and case presentations under the guidance of an attending neurologist. At the beginning and at the end of the elective, scores were obtained with a written and clinical skills state examination. The increase in scores was 6.3 % for the control group and 16.3 % for the intervention group.

### Types of bedside teaching

The question remains in what form bedside teaching should be given. One study evaluated two different bedside teaching methods compared with a control group when examining the cardiovascular system: demonstration and practice (DP) and collaborative discovery (CD) [44]. Both were done three times in two-hour sessions. In the DP group, the classical approach to bedside teaching was used. One (or a few) residents showed part of the clinical examination in front of the group and reported their findings. The teacher corrected or confirmed these findings and demonstrated the correct examination. The group as a whole practised afterwards to implement the demonstrated examination skills. In the CD group, all residents performed part of the examination and reported their findings afterwards. The teacher categorized these findings neutrally and highlighted consensus and disagreement. He then proposed methods to standardize the examination and let the residents do the examination again. This process was repeated until consensus was met within the group.

The evaluation of skills was done using an OSCE. The technical aspects of the learned skills increased in both groups: 12 % for the DP group and 10 % for the CD group, the equivalent being an increase of four and three correctly performed examination skills. Only the CD group showed a significant increase of 5 % in the finding of key clinical aspects (predefined perceptual criteria leading to the correct diagnosis) in the examination, compared with the control group. This is the equivalent of two more key clinical findings. No significant difference was found between the two bedside teaching strategies.

### Satisfaction regarding bedside teaching

The decline in bedside teaching in medical curricula is viewed as a loss because of its merits in teaching certain important aspects of medical reasoning and clinical skills. As discussed, a frequently heard reason for this decline is the *assumed* burden it puts on patients when participating in these bedside rounds. However, it appears that patients are generally very satisfied with bedside teaching. Simons et al. [54] measured heart rate, blood pressure and plasma norepinephrine levels of patients during bedside teaching rounds and found no indications of increased stress. Apart from this physiological data, patients report in 77–85 % of cases that they actually enjoy bedside teaching sessions [27, 42, 47]. Compared with conference room presentations, a study done by Lehman et al. showed that there was a trend among patients to be more favourable towards bedside teaching (although not statistically significant). Even though a statistical basis was missing, bedside teaching was not experienced more as a burden to patients [41].

Patients appear to enjoy bedside teaching and even report to obtain a better understanding of their disease. This conclusion not only goes for adult patients, but is also applicable in research from paediatric wards [40, 50]. One study looked at conference room presentations versus bedside teaching in a paediatric intensive care unit [40]. The parents of patients reported significantly more satisfaction with bedside teaching than teaching away from the bedside. They even rated the residents more competent when the case was presented at the bedside. The residents felt somewhat more uncomfortable when asked questions at the bedside, but in general they did not feel more discomforted with one of the two learning modalities.

Another study evaluated these factors in an outpatient paediatric clinic [50]. Overall, parent satisfaction was high, with no significant difference between the two groups. The attending physicians and residents preferred bedside teaching, especially because of the possibility to demonstrate more clinical skills and receive/give direct feedback. The attending physicians only noted a decreased level of comfort with the residents, and the residents reported this as well. Remarkably, this study showed no significant difference in time spent on the discussion of a case, either in the conference room or at the bedside.

Satisfaction scores of the students or residents and attending physicians appear to coincide with responses from patients. All students, interns and residents seem to think bedside teaching is an important way of learning clinical skills, but only 48 % report that they received enough of it in their medical education [42]. The majority of clinical teachers, as well as interns/residents, also think bedside teaching is effective in teaching physical examination skills, history taking and communication [42, 43].

### Solutions to overcome the decline in bedside teaching

A variety of strategies are proposed to provide some counterbalance to the increasing decline in bedside teaching. Some authors propose to reform the attitude of faculty regarding bedside teaching [49]. It seems that educational interventions can change the amount of time spent on bedside teaching (from less than 1 % to 41 % in one study) [33]. To cope with the increased workload for clinical staff, a shift of some educational tasks to residents and even to interns has been successful [29, 36]. Different educational tasks can be divided between varying competent groups in this manner. Finally, practical recommendations describe in more detail that bedside teaching should be structured well before, during and after the encounter, thereby reducing the risk of possible discomfort from the side of the patient, as well as learners and teachers [37, 43].

## Discussion

The discussed studies show a diverse representation regarding the added value of bedside teaching. Regarding the study by Favrat et al. [30]: although the authors were aware of the limitations of the study—especially the lack of a control group without the teaching intervention—it still shows a significant increase in correctly diagnosing patients on the basis of increased clinical experience. The relative increase is impressive (66 %), especially when considering the relatively low scores of the cardiologists involved in the study. The study does not speculate in which way these results could be extrapolated to other medical fields. It could be theorized that regarding the difficulty of cardiac auscultation in correctly diagnosing disorders, the training programme would be even more valuable for other (less difficult) areas of history taking and physical examination.

The same could be said for the study by Heckman et al. [35]. Although the absolute increase in test scores might once again seem little, it should be noted that the scores at the beginning were high (on average 75 % in both groups), which increases the difficulty of improvement. Especially the increase of 16 % in the intervention group is remarkable (they scored an average of 91 % after the elective). It could be speculated that with a lower starting score, the increase would have been even more substantial. A limitation was that the two groups were not randomized.

Regarding the type of bedside teaching provided, the study by Smith et al. [44] shows us a marginal (but significant) increase in key clinical findings when residents are working together in the diagnostic process compared with a control group. This marginal increase could be explained by the difficulty of the examined procedures (again cardiac murmurs) and the relatively intense and short teaching schedule (32 h shifts). A more structured, longer approach with less difficult parts of the physical examination might once again yield even better results.

The study by Sverdrup et al. [45] randomized two groups of students and found no difference in diagnostic skills in the end. This study has a lot of limitations, though, as the training course was short (only 4 h instead of the extended 5-month weekly training course in the study by Favrat et al.), the study population consisted of inexperienced third-year medical students and also no control group was used. Therefore, no conclusion can be made about the additional value of any of the two interventions.

A limitation of this review is the limited amount of evidence that is found in favour of bedside teaching. Randomized studies with the use of control groups are scarce in the already limited literature that exists on the subject. This is not surprising in a field which requires great efforts for proper investigation.

Most arguments regarding the subject are therefore based on rational assumptions and personal preferences, usually in favour of bedside teaching. But even though the evidence is limited, two empirical studies have provided relevant evidence for a beneficial effect of bedside teaching (in cardiology and neurology) [30, 35]. General history taking and physical examination skills are likely to show even more improvement if researched properly.

Furthermore, there seem to be few arguments *against* bedside teaching. A changing hospital and learning environment together with a greater technological reliance and other practical obstacles are frequently argued in the literature. But they are rather impediments to the bedside teaching modality, not real arguments directed at the rationale of bedside teaching as a possible successful educational option. Most authors share the opinion that these impediments do not outweigh the arguments and evidence in favour of bedside teaching and should therefore be adequately addressed. Only in this way can bedside teaching remain a valuable clinical teaching strategy.

## Conclusion

Bedside teaching seems to be gradually disappearing from medical curricula. As sophisticated diagnostic methods have reduced the need for physical diagnosis at the bedside, the *teaching* at the bedside runs the risk of being like a baby thrown out with the bathwater. Various reasons are given for this decline, including a changing hospital environment with increasing time constraints and discomfort on the part of the physicians. Bedside teaching is rationally necessary in learning certain clinical skills and evidence supporting the value of bedside teaching is found for different medical specialities (cardiology and neurology). Patients usually regard bedside teaching as enjoyable and not as a burden. Students, interns, residents and clinical teachers all generally appear to favour bedside teaching for the integration and learning of certain important clinical skills. From the literature, it follows that certain obstacles still remain, or even increase in the present day learning climate. Considering that bedside teaching is still valued by many involved in medical education, ways to overcome these obstacles should be found.

## Essentials

- Bedside teaching is declining in the medical curriculum. Reasons include an increased patient turnover in hospitals, the availability of high-quality diagnostic procedures other than physical diagnosis, and practical and personal impediments.
- Bedside teaching has been found to improve certain clinical skills in students and residents.
- Bedside teaching is still valued by patients, as well as students, residents and clinical teachers as a very useful teaching method.
- Because of its value for students/residents, patients and medical teachers, obstacles to bedside teaching should be overcome. Solutions vary from practical guidelines to using residents and interns as bedside teachers.
